# The Effect of Group Logotherapy on Meaning in Life and Depression Levels of Iranian Students

**DOI:** 10.1007/s10447-014-9225-0

**Published:** 2014-11-08

**Authors:** Somaye Robatmili, Faramarz Sohrabi, Mohammad Ali Shahrak, Siavash Talepasand, Mostafa Nokani, Mohaddese Hasani

**Affiliations:** 1 Department of Clinical Psychology, Allameh Tabataba’i University, Tehran, Iran; 2 Head of Counseling Center of Amir Kabir University of Technology, Tehran, Iran; 3 Department of Educational Psychology, Semnan University, Semnan, Iran; 4 Department of Psychiatry, Medical Sciences University, Arak, Iran; 5 Department of Psychology, Zahedshahr Branch, Islamic Azad University, Zahedshahr, Iran

**Keywords:** Logotherapy, Meaning in life, Depression, Iranian students

## Abstract

This paper identifies the effectiveness of group logotherapy in reducing depression and increasing meaning in life levels of university students in Iran. A randomized controlled trial was conducted with a pre- post- and follow-up test design. The instruments used were the *Purpose in Life* (PIL) test and the *Beck Depression Inventory* (BDI). Data were collected from 10 subjects in an experimental group and 10 in a control group. The experimental group participated in 10 sessions of group logotherapy, whilst the control group received no intervention. The mean scores for depression levels was significantly lower in the experimental group than in the control group and significantly higher in regard to meaning in life. Results suggest that group logotherapy has the potential to reduce depression levels and improve the meaning in life of university students.

## Introduction

Depression is a significant contributor to the global burden of psychological distress and affects people in all communities across the world (WHO 2012). The effects involve a depressed mood, a loss of interest or pleasure, decreased energy, feelings of guilt or low self-worth, disturbed sleep and appetite, and poor concentration (WHO 2012). Today, it is estimated to affect 350 million people, with a World Mental Health Survey conducted in 17 countries finding that, on average, about 1 in 20 people reported having an episode of depression in the previous year (WHO 2012).

Depression may have a number of distinct causes, such as a chemical imbalance, environmental pressures, interpersonal problems, intrapersonal difficulties, lack of meaning in one’s life, or a combination of these (Allen-Meares 1995). Many people experience their first onset of depression during college years. Unfortunately, many college students who have depression are not getting the help they need. They may not know where to go for help, or they may believe that treatment will not help. Others do not get help because they think that their symptoms are just part of the typical stress of college, or they worry about being judged if they seek mental health care (Eisenberg, Golberstein, and Gollust 2007).

The relative prevalence of depression in college students has also received much attention in the literature. Based on clinical diagnosis, depression in college students is more prevalent than in the general population—by some estimates about 11.8 % (14.3 % for females and 7.3 % for males), compared to about 5.2 % in the general population (American College Health Association 2006). Adverse outcomes include poor academic achievement, increased rates of substance use, co-morbid psychiatric conditions, and suicide (Moreno, Jelenchick, Egan, Cox, Young and et al. 2011).

During this stage of life, college students typically develop their self-identity and a sense of meaning and purpose in life. If they fail to find such purpose, they can readily lose confidence and can experience depression and a sense of meaninglessness. If they lack meaning and purpose in life, they are likely to experience existential emptiness, which involves a state of despair, in combination with depression, helplessness and emptiness (Frankl 1988).

Frankl (2006) asserted that the will to acquire meaning in life is a significant and universal human motive, the loss of which is characterized by boredom, hopelessness, depression, and the loss of the will to live. Yalom (1980) upheld that a human life without existential meaning can be very unsatisfactory and can result in major depression. Frankl (2006) suggested that the most difficult psychological issue facing people in the modern world is existential emptiness due to a lack of meaning in life, and he developed a therapeutic approach called Logotherapy to address this most challenging hurdle.

Logotherapy is a psychological, therapeutic treatment to address the root of such problems. It helps people appreciate their existence, gain liberty from emotional distress, and find meaning and purpose in their lives. Having meaning in life is regarded as being aware of the overriding goals of one’s life that add purpose to everyday living and is a primary motivational force in humans (Frankl 1988).

Group logotherapy appears to be uniquely positioned to deal effectively with existential concerns. Yalom (1985) offered an excellent explanation of this existentially normalizing group process. More specifically, he noted: “the group configuration is not ‘you,’ the therapist, and ‘they,’ the dying; but it is we who are dying, we who are banding together in the face of our common condition” (p. 34). Group logotherapy is, therefore, an attempt to leverage the therapeutic value of exploring issues of existence by capitalizing on the existentialism imbedded in the group process (Somov 2007).

Studies on the application of group logotherapy with university students have been few, in general and in regard to depression and issues of meaning in life, and few community mental health centers provide logotherapy in Iran. Therefore, the purpose of this experimental study was to test the effect of a group logotherapy approach with university students in Iran experiencing levels of depression and meaningless in life.

The following hypotheses were tested: (a) that participation in group logotherapy would improve university students’ sense of meaning in life and reduce depression levels, and (b) that the effects of such group logotherapy would be sustained at a one-month follow-up point.

## Methodology

### Participants and Procedures

The study followed a pre-test, post-test design with a one-month follow-up involving both an experimental and a control group. The participants of this study were students of the Shahid Chamran University in Ahvaz City, Iran. Approval to undertake the study was provided by the research committee in the Department of Clinical Psychology.

From all the students studying for a Bachelor of Science degree, five hundred and six (506) students were selected following standard sampling procedures (Kerjcie and Morgan 1970). Following assurances about confidentiality, the participants provided informed consent to be involved and completed instruments assessing their depression levels (Beck’s Depression Inventory) and their sense of meaning in life (Purpose in Life). One hundred and twenty-one (121) students among the 506 were found to have significant levels of meaninglessness and depression (i.e., those with scores of 100 and below in the PIL, and 13 and above in the BDI). Finally, from that group of 121, 20 students were randomly assigned to the experimental (10 students) and control (10 students) groups. The former, took part in 10 × 1 h weekly group logotherapy sessions at the university’s counseling centre, whilst the latter were placed on a waiting list for individual counseling. Post-test assessments were conducted for each group following the 10 week program period and at a one-month follow-up point.

### Intervention

The intervention with the experimental group involved a logotherapy approach and emphasized finding value in an individual’s life in order to attain meaning. The fundamental structure of the Logotherapy program undertaken is shown in Table 1. The program was designed to 1) help participants clarify values that were particularly meaningful to them, 2) set reasonable goals, 3) assure that the goals would actualize the participants’ meaningful values, 4) set practical plans to achieve the goals, 5) identify participants’ assets and deficits that would affect their attempts to achieve their goals, and 6) intentionally incorporate the assets and deficits into the plans to achieve the goals to actualize the values.

**Table 1:** Fundamental structure of the Logotherapy program

Sessions Outline
1. Introduction. Logotherapy basic information and intended course of group logotherapy. Explanation of Values Awareness Technique. Group exercise: “What I want to be”. Homework: beginning exploration of Creative Values.
2. Clarification of Creative Values. Discussion about progress/difficulties with homework. Distribution of Values Worksheet. Group exercise: satisfying achievements. Homework: completing exploration of Creative Values.
3. Clarification of Experiential Values. Discussion about progress/difficulties with homework. Group exercises: Recent Events, Positive Persons, Artistic Expressions. Homework: completing exploration of Experiential Values.
4. Clarification of Attitudinal Values. Discussion about progress/difficulties with homework. Group exercise: Wise Sayings, Taking a Stance, My Obituary. Homework: completing exploration of Attitudinal Values.
5. Focus on Goals. Discussion about progress/difficulties with homework. Elaboration: Values Hierarchy. Group exercise: Setting Goals. Group exercise: another Perspective on Goals.
6. Fitting goals with values. Discussion about progress/difficulties with homework. Analyzing goals for fit with personal values. Homework: Participants analyze a variety of their goals by the method discussed during this current session. Participants should become aware of any leftover (unmarked) values for short-term goals, for intermediate goals, and for long-term goals.
7. Setting new goals. Group discussion about Homework results and insights. Setting new goals for leftover values. Homework: Using the method described in this session, participants set a new short-term goal, a new intermediate goal, and a new long-term goal. Participants evaluate each new goal against each value in their Values Hierarchy using the process demonstrated in the previous session. If leftover values still remain after the new goals are compared against the Values Hierarchy, then additional goals are set until no leftover values remain.
8. Planning for goal achievement. Goal achievement outline (the goal should be measurable, attainable). Discussion: Ideas related to the topic of establishing plans to achieve goals. Homework: using the method described in this session, participants set goal achievement plans for one short-term goal; a goal achievement plan for one intermediate goal; and a goal achievement plan for one long-term goal.
9. Current status analysis. Group discussion: homework results and insights; each participant shares the three goals for which they have established plans. Group exercise: assets and deficits. Group discussion: it is important to know our deficits as well as assets because once we are aware of them we are then in a position to make the choice to change or not change. Homework exercise: incorporating assets and deficits into plans.
10. Summary and Critique. Participants share examples of how they will incorporate their assets and deficits into their plans to achieve their goals. Summarization: what the course of the group has been; any comments about the group. Group discussion: any changes participants see in themselves as a result of attending the group logotherapy. Critique: three best and three worst components of the group logotherapy; three suggestions for changes that could improve the group logotherapy. End.

During the initial sessions, the focus was on clarifying participants’ meaningful values. This was accomplished by means of the Values Awareness Technique (VAT). Briefly, the VAT consists of a series of exercises, each of them involving a three-step format: i) Expanding Conscious Awareness - participants are instructed to observe their lives from a different perspective, ii) Stimulating Creative Imagination - participants are instructed to “brainstorm” many possible values that could underlie the results from step-1, iii) Projecting Personal Values - participants are instructed to select, out of the many step-2 results, those particular values that underlie their particular responses in step-1. Following several of these three-step exercises, participants were instructed to identify the values that they repeatedly selected during the exercises. After clarification of participants’ individual values, the focus was on goals - fitting goals with the values, setting new goals for values, planning for goal achievement, and undertaking ongoing current status analysis in regard to the process.

The notion of values within the approach is subcategorized into three main areas: creative, experiential, and attitudinal. Creative values are reached through acts of creating or producing something. Experiential values are actualized when a person experiences something through sight, touch, smell, or hearing. Finally, attitudinal values are reserved for individuals who cannot, for one reason or another, have new experiences or create new things. Thus they find meaning through adopting a new attitude that allows “suffering with dignity”.

For all of these classes of values, it is considered to be because of one’s sense of responsibility that one pursues such values and consequently experiences a meaningful life. It is through the realization that one is the sole being responsible for rendering life meaningful that values are actualized and life becomes meaningful (Feldman and Snyder 2005).

### Instruments

The instruments chosen for the study were the Purpose in Life (PIL) Test to measure meaning in life, and the Beck Depression Inventory to measure depression levels. The former was designed by Crumbaugh and Maholick (1964) to operationalize Frankl’s ideas and to measure an individual’s experience of meaning in life. It is a 20-item scale and has been shown to have good reliability (Seeman 1991; Zika and Chamberlain 1992). Each item is rated on a 7-point scale from 1 to 7, with total scores, therefore, ranging from 20 (low purpose) to 140 (high purpose). Raw scores of 113 and above are typically interpreted as high purpose, scores of 92–112 reflect moderate levels of purpose, and scores of 92 and below suggest a lack of life purpose (Crumbaugh and Maholick 1964).

High scorers on the PIL scale are seen to have relevant goals and a sense of direction in life. They feel that there is meaning to their life, both currently and in the past, they hold beliefs that give life purpose, and they have meaningful aims and objectives for living. Low scorers are seen to lack a sense of meaning in life, have few goals, lack a sense of direction, do not see purpose in their past, and do not have meaningful outlooks on life (Ryff and Keyes 1995). This instrument has been translated into Persian for use in Iran by Rahiminezhad, Kazemi, Farahani and Aghamohamadi (2011) with a Cronbach alpha of .92, and the Iranian version of the PIL test was used in this study.

The Beck Depression Inventory (BDI) was created in 1988 by Aaron T. Beck (Beck, Steer, and Garbin 1988). It is a 13-item self-report inventory that addresses depressive symptoms. Each item is rated on a 0–3 scale, with 3 indicating the most severe symptoms, and involves a total score ranging between 0 and 39. The BDI-13 has good internal consistency and concurrent validity (Segal, Coolidge, Cahill, and O’Riley 2006; Steer, Rissmiller, and Beck 2000; Beck, Steer, and Garbin 1988). This instrument has been translated into Persian for use in Iran by Dadsetan and Mansour (1990), with a Cronbach alpha of .87, and the Iranian version of the BDI was used in this study.

### Statistical Analysis

Data were analyzed by the use of SPSS/PC 16.0. The analyses undertaken involved descriptive statistics utilizing means and standard deviations for both meaning in life and depression scores in both the experimental and control groups in pre-test, post-test and follow-up assessments, and MANCOVA procedures for assessing homogeneity and differences between the two groups.

## Results

Table 2 shows the within-group means and standard deviations of the meaning in life and depression level scores in the experimental and control groups at pre-test, post-test and follow-up points. As is evident, the mean scores of the experimental group revealed distinct positive changes in meaning in life scores and also negative changes in depression levels at post-test and follow-up, compared to pre-test scores. Such a change was not observed in the control group.

**Table 2:** Group scores for meaning in life and depression levels

	Experimental				Control			
	Depression		Meaning in life		Depression		Meaning in life	
	Mean	SD	Mean	SD	Mean	SD	Mean	SD
Pre-test	14.00	2.49	92.00	16.49	14.30	3.48	97.95	16.71
Post-test	6.50	2.01	120.95	13.52	15.75	3.07	100.00	15.35
Follow-up	4.70	2.27	127.95	12.99	16.35	2.68	92.80	16.98

**Fig. 1:**
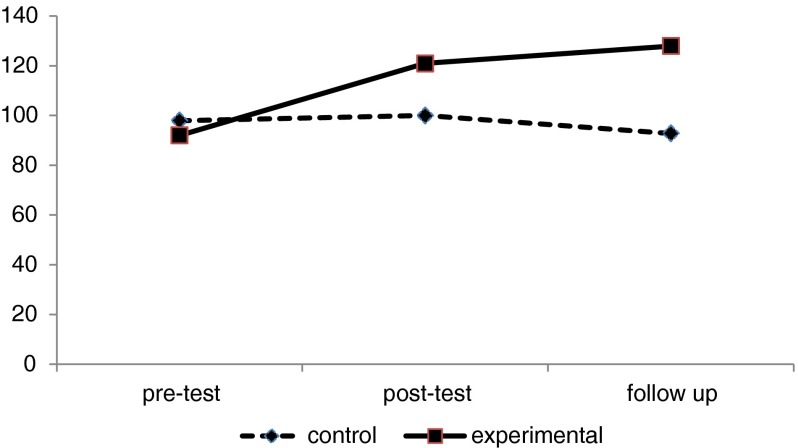
Mean scores of meaning in life

It was assumed that group logotherapy had a significant effect on meaning in life and depression levels for the experimental group. For testing this hypothesis, we examined between-group differences in relation to the dependent variables of meaning in life and depression levels by performing a MANCOVA test. First, we used pre-test scores as covariates and tested post-test scores in the experimental and control groups. There was a significant difference between the two groups in the dependent variables (Willks’ Lambda = .04, *F* = 420.5, *p* < .001). The post-hoc tests showed that the mean score for the meaning in life variable was significantly higher in the experimental group than in the control group (*F* = 290.48, *p* < .001) (see Fig. 1).

**Fig. 2:**
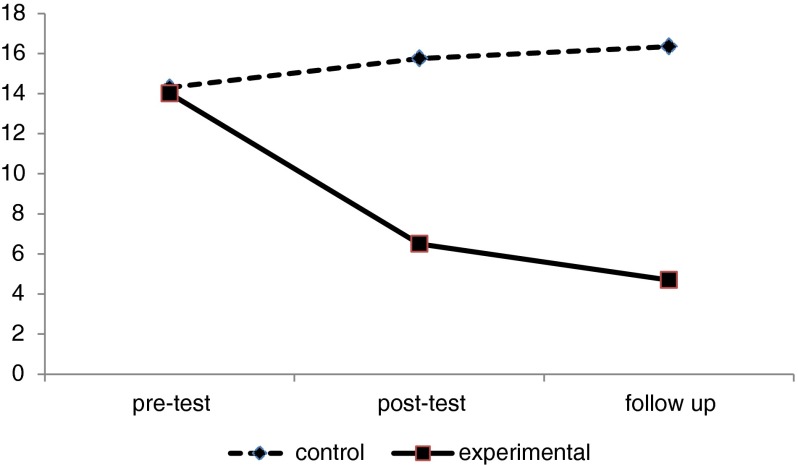
Mean scores of depression

In addition, post-test analysis showed that the depression level mean was significantly lower in the experimental group than in the control group (*F* = 198.69, *p* < .001) (see Fig. 2). Therefore, it was assumed that logotherapy had a significant effect on meaning in life scores and as well depression levels.

Also, changes in scores of meaning in life and depression level variables were examined for both groups after a one-month follow-up. Again, pre-test scores were used as covariates and there was a significant difference between the two groups in both dependent variables (Willks’ Lambda = .05, *F* = 323.4, *p* < .001). The post-hoc analysis showed that the mean score for the meaning in life variable was significantly higher in the experimental group than in the control group (*F* = 402.48, *p* < .001). In addition, the post-hoc tests showed that the mean score for depression levels was significantly lower in the experimental group than in the control group (*F* = 262.30, *p* < .001). Therefore, the apparent effect of logotherapy on both meaning in life scores and depression levels was stable after a one-month follow-up.

## Discussion

Before the administration of the Logotherapy program, all of the participants in both groups (experimental and control) were assessed with having a lowered sense of meaning in life and high depression levels. This was in line with Frankl’s (2006) description of people who would be experiencing what he termed an ‘existential vacuum’ or a general sense of meaninglessness, which involves a state of despair, in combination with depression, helplessness and emptiness (Frankl 1988).

Logotherapy, however, views individuals as having the potential to transcend their environments and the freedom and responsibility to make choices that are conducive to growth despite their circumstances. Logotherapy posits the following: (a) a primary and basically subconscious motivation in human existence is a “will to meaning,” and (b) meaninglessness, depression and other pathology often results when individuals are unable to identify and pursue a worthy sense of meaning (Brown and Romanchuk 1994; Frankl 1967; Guttmann 1996; Lantz and Alford 1995).

The experience of young people in regard to meaninglessness is well illustrated by Blair (2004) as he examined the principles of logotherapy among some older adolescents who perceived that their lives were devoid of meaning or who were facing difficult challenges and trying to make sense of those challenges. He observed the feeling of being lost among conflicting demands was especially true among youth who had not yet developed a clear sense of their own identities. These youth recurrently conformed to the values of their peers, even when these values were in opposition to their own personal beliefs (Pellebon and Anderson 1999). As a consequence of acting outside of their own value system, even when they are not fully aware of what that value system entails, such youth often feel in conflict with themselves and frequently manifest symptoms of meaninglessness, depression or other mental health problems. However, when given the opportunity to discuss and clarify their values and goals, they gain self-awareness and a clearer sense of identity and adhere to a personal value system, with the result being that their meaninglessness and depressive symptoms lessen.

The data in Table 2 show the results of pre-, post- and follow-up assessments of both experimental and control group participants in this regard. There are some obvious reasons for the lack of improvement in the control group’s post-test and follow-up mean scores. The group was on a waiting list for counseling and did not receive any treatment, so their ‘existential vacuum’ identified in the initial assessments persisted. The members of the experimental group, on the other hand, were assisted to be able to exercise the most important freedom of all: the freedom to determine one’s own attitude and spiritual well-being.

The findings suggest that the Logotherapy program had a positive effect on the depression levels and the general sense of meaninglessness in the participants of the experimental group. These results seem to affirm what Fabry (1994) observed, that people were able to value what was given to them, and what they valued guided their search for meaning and simplified their decision-making. Also, this result is clearly similar to Blair (2004), when he defined assisting meaningless and depressed adolescents in discovering and pursuing meaning is facilitated by helping them work through the following steps: (a) establishing the therapeutic relationship; (b) increasing insight regarding identity, values, and goals; (c) reframing meaninglessness and depression; (d) discovering meaning within the meaninglessness and depression; and (e) pursuing the fulfillment of meaning.

Going into greater depth, a crisis of meaning was experienced by participants. Frankl stated, “In the past, nothing is irretrievably lost” (Frankl 2006, p. 151). The accuracy of this thought was clearly realized by the participants of the experimental group during their first therapeutic session wherein they were encouraged to explore in the Group exercise: “What I want to be’ and an exploration of creative values. By the fourth session, through clarification of creative, experiential and attitudinal values, they were able to realize and appreciate the unique pattern of their personal values. This is based on what Frankl believed, that meaning could come through what we give to life and what we take from the world (Frankl 2006). After helping the participants see the uniqueness of their personal values, they were encouraged to focus on goals - fitting goals with values, setting new goals for values and planning for goal achievement. Finally, participants were helped to make an active resolution to persevere in life despite the presence of the difficulties.

This study was limited to university students; therefore, it cannot be generalized to other ages and groups. Also, there was no consideration given to gender differences. As such, it is suggested that future research investigates and compares the effectiveness of such a programme of meaning-finding on each gender group.

## Conclusion

This group logotherapy program for university students in Iran with depressive symptoms and a low sense of meaning in life was found to be effective in reducing depression levels and enhancing a sense of meaning in life. Life without meaning is empty. Meaning is available to each individual under any circumstances because a person has the freedom of will and the will to define meaning.

Logotherapy potentially offers long-term relief from the underlying meaninglessness that is experienced by some university students. Though the existing empirical data are sparse, it is clear that Logotherapy deserves further research, not just as an adjunctive treatment, but, rather, as a structured and effective therapy for the treatment of meaninglessness and depression. Irvin Yalom (2005, p. 230) once stated that humans struggle as a result of being a “meaning-seeking creature in a meaningless universe” (p. 230). This is an important dilemma that should be addressed in therapy with clients experiencing a sense of meaninglessness. Mental health professionals, regardless of theoretical allegiance, should engage with their clients and empower them with the responsibility to seek and create meaning.
